# Cytokine Dynamics in Severe COVID-19 vs. Influenza A Elderly Patients: A Prospective Comparative Study

**DOI:** 10.3390/ijms27031463

**Published:** 2026-02-01

**Authors:** Mihai Aronel Rus, Adina Huțanu, Daniel Corneliu Leucuța, Violeta Tincuța Briciu, Monica Iuliana Muntean, Angela Ionică, Mihaela Sorina Lupșe

**Affiliations:** 1 Department of Infectious Diseases, “Iuliu Hatieganu” University of Medicine and Pharmacy, Iuliu Moldovan Street, no 23, 400348 Cluj-Napoca, Romania; mihai.rus@umfcluj.ro (M.A.R.);; 2 Department of Laboratory Medicine, George Emil Palade University of Medicine, Pharmacy, Science, and Technology of Târgu Mureș, 540142 Targu Mures, Romania; 3 Advanced Medical and Pharmaceutical Research Center, George Emil Palade University of Medicine, Pharmacy, Science, and Technology of Târgu Mureș, 540136 Targu Mures, Romania; 4 Department of Medical Informatics and Biostatistics, Iuliu Hatieganu University of Medicine and Pharmacy, Pasteur Street, no 6, 400349 Cluj-Napoca, Romania; 5 Clinical Hospital for Infectious Diseases, Iuliu Moldovan Street, no 23, 400348 Cluj-Napoca, Romania

**Keywords:** COVID-19, influenza A, cytokines, Luminex

## Abstract

COVID-19 and influenza A (FluA) cause severe respiratory infections in elderly patients, with cytokine dysregulation playing a central role. Direct comparative data in older adults remains limited. We aimed to characterize cytokine dynamics and their prognostic value in hospitalized elderly patients with COVID-19 vs. FluA. We performed a prospective cohort study including adults ≥ 60 years hospitalized with respiratory failure due to COVID-19 or FluA between March 2023 and March 2024. Serum IL-1β, IL-6, IL-10, IL-17A, IL-34, MCP-1, and CXCL10 were measured on Day 1 and Day 5 of hospitalization using Luminex^®^. Cytokines and associations with non-invasive ventilation (NIV) were assessed by ROC analysis and multivariate logistic regression. 83 patients were included (39 COVID-19, median age 79 years; 44 FluA, median 77 years). At Day 1, COVID-19 exhibited significantly higher IL-6, IL-10, and CXCL10; FluA showed an attenuated cytokine response. At Day 5, cytokines declined in both groups. Baseline IL-6 independently predicted NIV (adjusted OR 3.02), whereas higher MCP-1 was associated with reduced NIV requirement. Early cytokine differences between COVID-19 and FluA are evident in elderly patients, but values converged by Day 5. IL-6 remains an informative early predictor of respiratory deterioration; MCP-1 may reflect a regulated innate response.

## 1. Introduction

COVID-19 and influenza A (FluA) are acute viral respiratory infections and among the most frequent causes of hospitalization for respiratory illnesses in adult patients [1,2]. The two infections tend to overlap regarding clinical manifestations; both display epidemic patterns with seasonal increases in incidence and inter-epidemic decline [3], but display distinct immunopathological signatures [4]. Understanding the host immune mechanisms that underlie these divergent outcomes remains essential for identifying prognostic markers and guiding therapeutic interventions.

Innate immunity constitutes the first line of defense against both viruses, yet the timing, magnitude, and regulatory balance of these responses diverge. In FluA infection, early recognition of viral RNA by RIG-I, TLR7/8, and the NLRP3 inflammasome triggers a prompt release of type I interferons and proinflammatory cytokines such as IL-1β, IL-6, and TNF-α, along with chemokines like CXCL10 that recruit monocytes and NK cells to the infected airway [5]. The parallel induction of regulatory mediators, including IL-10, and Th17-related cytokines such as IL-17, contributes to immune modulation and epithelial repair, resulting in a balanced antiviral and inflammatory response that promotes viral clearance while limiting tissue damage [6]. In contrast, SARS-CoV-2 primarily engages MDA5 and the cGAS–STING pathways but frequently suppresses or delays interferon signaling through multiple antagonistic proteins (e.g., NSP1, ORF6, ORF8), resulting in inadequate early control followed by excessive cytokine and chemokine production [7]. This dysregulated response—characterized by markedly elevated IL-6, IL-8, IL-10, and CXCL10, neutrophil activation, and NK-cell exhaustion—contributes to the hyperinflammatory phenotype and tissue injury observed in severe COVID-19 [8].

Cytokine signaling represents a crucial interface between innate and adaptive immunity in both infections, orchestrating viral clearance, inflammation, and tissue repair. Among them, IL-6 is a central effector of the acute-phase response, promoting CRP synthesis, endothelial activation, and lymphocyte differentiation [9], and has been consistently associated with respiratory failure and mortality in severe COVID-19 [10]. Interleukin-10, an anti-inflammatory cytokine produced by monocytes and regulatory T cells, can limit tissue damage; for instance, in influenza, IL-10 signaling has been shown to interfere with antiviral defense and modify Th17/antibody responses, highlighting its time-sensitive role in viral pneumonia [5]. Interleukin-1β, produced downstream of inflammasome activation, and IL-17A, a key mediator of Th17 responses, both amplify neutrophil recruitment and contribute to epithelial damage and lung injury in severe viral pneumonia [5,11]. IL-34, a macrophage colony-stimulating factor receptor ligand, supports monocyte survival and tissue regeneration, while chemokines such as MCP-1 (CCL2) and CXCL10 (IP-10) regulate leukocyte recruitment to the infected lung and are closely linked to disease severity in COVID-19 [6,8]. Prior studies have reported higher systemic levels of IL-6, IL-10, and CXCL10 in COVID-19 than in FluA, yet most relied on single-time-point sampling from early pandemic waves [4]. Accumulating evidence suggests that Omicron-lineage SARS-CoV-2 infections are generally associated with attenuated systemic inflammation and reduced clinical severity compared with earlier variants, although severe disease remains frequent in older adults. The temporal dynamics of cytokine responses, especially during the Omicron era, remain insufficiently characterized and may hold the key to understanding the shifting balance between hyperinflammation and immune regulation.

Notably, severe COVID-19 and FluA disproportionately affect older adults, in whom immunosenescence and chronic low-grade inflammation (inflammaging) impair antiviral defense and amplify cytokine responses [12,13]. Given the context, characterizing the temporal dynamics of cytokine responses in this vulnerable population is essential for understanding disease progression and prognosis. Moreover, despite extensive research on cytokine responses in COVID-19 and influenza A, direct comparative data based on paired, longitudinal cytokine measurements in elderly patients remain scarce, particularly during the Omicron era. Therefore, in this study, we aimed to assess Day 1–Day 5 cytokine dynamics of IL-1β, IL-6, IL-10, IL-17A, IL-34, MCP-1, and CXCL10 in older adults hospitalized with COVID-19 or influenza A and to explore their prognostic value for respiratory deterioration and clinical outcomes.

## 2. Results

### 2.1. Cohort Characteristics

A total of 83 patients were included in the analysis: 44 (53%) with severe FluA and 39 (47%) with severe COVID-19. According to the inclusion criteria, all participants’ ages were ≥60 years old, the COVID-19 cohort being older (median age of 79 years old vs. 77 years old for FluA). The female sex was significantly more prevalent in the FluA group, 79.5% vs. 46.15% in COVID-19, *p* = 0.002. The median age-adjusted Charlson comorbidity index (ACCI) and length of hospitalization were identical for both groups. We noticed that COPD was more frequent for FluA patients, with statistical significance. Hemiplegia was recorded in 5 COVID-19 patients and was not observed in the FluA group (*p* = 0.02). Detailed cohort characteristics are represented in Table 1.

All baseline laboratory parameters were very similar between the groups, without statistically significant differences. Related to imaging findings, COVID-19 patients had significantly more frequent ground-glass opacities (53.85% vs. 25%, *p* = 0.007) and more frequent bilateral consolidation (30.7% vs. 15.9%, *p* = 0.108), while FluA patients had more frequent interstitial involvement, without statistical significance. Detailed laboratory and imaging results are presented in Table 2.

### 2.2. Outcome

As per the inclusion criteria, all patients had respiratory failure requiring oxygen supplementation on admission. NIV was more frequent in the FluA group (77.2% vs. 61.4%, *p* = 0.187), whereas invasive ventilation was a rare occurrence in both groups of this study. ICU admission was more often in the COVID-19 group, but the number of patients was small and without statistical significance. There were 3 deaths among COVID-19 patients and 2 deaths in the FluA group. Apart from respiratory manifestations, acute renal failure was the most frequent complication, and it was more frequent in FluA patients (31.8% vs. 15.3%, *p* = 0.081). Other analyzed complications, such as pulmonary embolism, stroke, and atrial fibrillation, were rare. All patients in the COVID-19 group and 43 of 44 patients in the FluA group received antibiotic therapy during hospitalization. Antiviral treatment was administered to all patients according to the standard of care. FluA patients received oseltamivir, and COVID-19 patients received remdesivir. Detailed outcomes and complications are presented in Table 3.

### 2.3. Cytokines Analysis

Cytokine analysis revealed that at Day 1 sampling, patients with COVID-19 displayed higher median values and also higher maximum values for IL-6, CXCL10, and the anti-inflammatory cytokine IL-10, compared to those with FluA. MCP-1 median concentrations were also higher in the COVID-19 group, but the maximum individual values were observed in the FluA group. IL-17A showed low and comparable values in both cohorts. At Day 1, only IL-6 levels differed significantly between COVID-19 and FluA (*p* = 0.009), with higher levels in the COVID-19 cohort, using the Mann–Whitney U test and a statistical significance threshold of *p* < 0.05. At Day 5 sampling, concentrations of all interleukins decreased substantially. The median values of IL-6 at Day 5 were, interestingly, 3.7 times lower for both infections. IL-10, MCP-1, and CXCL10 also showed similar patterns of fold decrease in the median values. No statistically significant differences in the assessed interleukins were found between the two groups at Day 5. Among the seven cytokines initially measured, IL-1β and IL-34 showed very low or non-quantifiable concentrations in the majority of samples and were therefore excluded from group comparisons and correlation analyses.

Cytokine values at Day 1 and Day 5 are presented in Table 4.

### 2.4. Cytokines and Non-Invasive Ventilation

To explore whether early cytokine levels could help identify patients at increased risk of requiring NIV, we performed receiver operating characteristic (ROC) analyses of the biomarkers measured at Day 1 and Day 5. In our cohort of elderly patients with respiratory failure at admission, most cytokines demonstrated modest discriminative ability, with AUC values generally ranging between 0.50 and 0.60. Among the Day 1 measurements, IL-6 and the IL-6/IL-10 ratio yielded the highest AUC values (0.603 and 0.618, respectively), although these remained within a limited predictive range. Interleukin-10 and CXCL10 at baseline showed minimal classification capacity. Notably, MCP-1 measured at Day 5 reached the highest overall AUC (0.686), with a cut-off of 251 pg/mL providing a balanced sensitivity (66%) and specificity (71%), whereas other Day 5 cytokines remained only weakly informative. Given these findings and the modest AUC values observed, ROC-derived thresholds were used not as diagnostic classifiers but solely as exploratory, data-driven cut-off points to facilitate dichotomization of cytokine levels in subsequent multivariate analyses. The complete ROC metrics are presented in Table 5.

To further assess whether cytokines were independently associated with the need for NIV, we constructed multivariate logistic regression models adjusted for infection type (COVID-19 vs. FluA). Using ROC-derived thresholds, Day 1 IL-6 emerged as the only baseline cytokine significantly associated with NIV requirement: IL-6 concentrations ≥ 8.7 pg/mL were linked to three-fold higher odds of needing NIV (adjusted OR 3.02, 95% CI 1.06–9.53). The IL-6/IL-10 ratio at baseline showed a similar trend but did not reach statistical significance. In contrast, MCP-1 demonstrated a protective association, with elevated values at Day 1 (OR 0.32, 95% CI 0.11–0.88) and Day 5 (OR 0.25, 95% CI 0.09–0.67), both associated with a lower likelihood of NIV use. The IL-6 at Day 5, IL-10, CXCL10, IL-17A, and dynamic cytokine changes did not demonstrate significant associations with respiratory deterioration. The infection type itself was not independently associated with NIV in any model. Detailed multivariate results are shown in Table 6.

We next explored whether cytokine trajectories were coordinated during early hospitalization by assessing correlations between percentage changes from Day 1 to Day 5 (Δ). In COVID-19, ΔIL-6 correlated with ΔMCP-1 (0.52; *p* < 0.001), and ΔIL10 showed a strong correlation with ΔCXCL10 (0.95; *p* < 0.001), indicating that several inflammatory mediators tended to change in the same direction. In contrast, cytokine co-variation in FluA was more limited. The strongest association was observed again between ΔIL-10 and ΔCXCL10 (r = 0.99; *p* < 0.001), while correlations involving IL-6 and IL-17A were weaker and did not reach statistical significance. These patterns suggest that COVID-19 is characterized by a more synchronized evolution of multiple cytokine pathways, whereas FluA displays a narrower profile of coordinated cytokine changes. Full correlation matrices are presented in Supplementary Tables S1 and S2.

## 3. Discussion

In this prospective cohort of elderly patients hospitalized with COVID-19 or FluA and respiratory failure at admission, we identified distinct early cytokine signatures between the two infections, despite a relatively homogenous cohort with comparable demographics, Charlson comorbidity index, and baseline laboratory results. At Day 1, patients with COVID-19 displayed higher serum concentrations of IL-6, IL-10, and CXCL10, whereas FluA showed a more attenuated systemic cytokine response. By Day 5, however, cytokine levels declined markedly across both groups, and between-group differences were no longer relevant.

The significantly higher IL-6 concentrations observed in COVID-19 are consistent with extensive evidence identifying IL-6 as a central mediator of SARS-CoV-2–induced inflammation, respiratory deterioration, and mortality, as it has been repeatedly demonstrated in cytokine storm analyses and severity prediction models [10,14,15,16]. Other comparative studies reported higher IL-6 levels in hospitalized COVID-19 patients compared to FluA [4,17], but most cytokine comparisons between the two infections were conducted before the emergence of Omicron. In FluA, IL-6 is also a key inflammatory mediator, but its release tends to be more tightly linked to direct epithelial injury and early alveolar damage rather than sustained systemic inflammation [6]. The comparatively higher IL-6 levels in COVID-19 likely reflect the combination of delayed interferon signaling and prolonged monocyte–macrophage activation characteristic of SARS-CoV-2 infection, which amplifies systemic cytokine release beyond the more localized and rapid inflammatory response typically triggered by FluA [18]. In our cohort, IL-6 at Day 1 remained the only cytokine independently associated with the need for NIV. This reinforces IL-6 as a key element of regulating inflammation and a reliable prognostic cytokine.

Although IL-10 concentrations were higher in COVID-19 at baseline, IL-10 did not predict the need for NIV in our cohort. IL-10 primarily regulates macrophage and dendritic-cell activity by suppressing pro-inflammatory signaling and modulating interferon-related pathways [19]. At high levels, IL-10 is generally interpreted as a marker of early “immunoparalysis,” and several studies have associated markedly elevated IL-10 values with dysregulated host responses and severe COVID-19 [16,20]. Previous comparative analyses show that both viruses induce IL-10, but SARS-CoV-2 tends to elicit a more prominent IL-10 response than FluA, a pattern interpreted as early immune dysregulation [16]. In our cohort, however, IL-10 declined substantially by Day 5 in both infections, showing that the early elevation was transient and not associated with subsequent clinical deterioration in our population of elders.

Similarly to IL-10, CXCL10 concentrations were higher in the COVID-19 group, but they did not predict the need for NIV in our study. CXCL10 is an interferon-stimulated proinflammatory chemokine that orchestrates the recruitment of activated T cells and NK cells to infected lung tissue, and SARS-CoV-2 is known to induce marked CXCL10 upregulation through IFN-dependent pathways [21]. Other comparative analyses have reported stronger CXCL10 induction in COVID-19 than in FluA, a feature attributed to SARS-CoV-2–mediated dysregulation of interferon signaling [16]. High CXCL10 levels have been associated with progression to severe disease and ICU admission in COVID-19 [22], but these associations are less consistent in elderly populations, where interferon responses are blunted and more heterogeneous. In our cohort, CXCL10 levels declined substantially by Day 5 and showed no prognostic value, suggesting that interferon-driven chemokine activity was transient and insufficient to discriminate early respiratory deterioration in older adults hospitalized during the latter SARS-CoV-2 waves.

Among the cytokines evaluated in our study, MCP-1 at Day 1 showed an unexpected association: higher MCP-1 concentrations were linked to a lower likelihood of requiring NIV. MCP-1 (CCL2) is typically regarded as a proinflammatory chemokine [23]. Chen et al. [24] found an association between MCP-1 elevation and disease severity in the first wave of COVID-19, but the MCP-1 values are higher than in our cohort. Polese et al. [25] also found that high MCP-1 values are associated with risk of ICU admission and death in a pre-Omicron cohort. Korobova et al. [26] found that patients with the Omicron variant had lower MCP-1 values than previous waves and a very similar median value to our cohort, but the study did not assess its prognostic role. Also supporting the idea of a more attenuated response in Omicron patients, another study that recruited patients during an Omicron wave, by Cheng et al. [27], found no statistically significant difference between MCP-1 values in severe vs. non-severe patients. Interestingly, fewer studies assessed this association in FluA. Davey et al. [28] found in an older FluA cohort that MCP-1 values are higher than in our cohort for patients with disease progression and death. Therefore, we hypothesize that this may be explained by the fact that we recruited COVID-19 patients with the Omicron variant, which exhibited a more blunted immune response, and especially resides in the fact that we had an old cohort, with the immune response influenced by immunosenescence. Nonetheless, in a regulated immune response, MCP-1 elevation plays a favorable role [29]. Although exploratory due to sample size, these findings suggest that MCP-1 trajectories may have distinct prognostic implications in aging populations and deserve further investigation. The divergent associations observed for IL-6 and MCP-1 indicate that these mediators reflect distinct inflammatory processes in severe viral pneumonia. Both are pro-inflammatory mediators; IL-6 is widely recognized as a marker of systemic inflammatory amplification and endothelial stress [10], whereas MCP-1, a chemokine, primarily regulates monocyte recruitment and immune cell trafficking [23]. Although vascular complications such as pulmonary embolism or stroke were rare and could not be formally analyzed, the diverging behavior of MCP-1 compared with IL-6 supports the concept that not all inflammatory mediators convey equivalent inflammatory or vascular risk [24,25].

Despite a more elevated systemic cytokine activation observed in the COVID-19 group, FluA required NIV more frequently. This finding is consistent with other previous reports during Omicron-dominant periods, where FluA patients needed NIV more frequently [3,17]. We found support for this divergence in the literature showing that FluA tends to cause a pronounced alveolar injury and parenchymal damage, leading to more severe hypoxemia. The review by Iwasaki & Pillai [5] and the analysis by Guo & Thomas [6] described how FluA triggers rapid activation of alveolar macrophages, intense NLRP3 inflammasome signaling, and direct epithelial cytopathic effects—processes that produce acute parenchymal damage. Moreover, the inflammatory response of COVID-19 patients is less severe during the Omicron wave than in previous waves [30], although still higher than in FluA in our study. Overall, these findings are consistent with the possibility that, in older adults, FluA-related respiratory deterioration is more closely linked to lung parenchymal injury, while COVID-19 retains a comparatively stronger systemic inflammatory component, even during the Omicron era. In other words, the distinction in NIV requirements may reflect not only disease severity but also whether inflammation remains predominantly lung-confined or becomes systemic, providing a framework for interpreting the observed cytokine patterns across the two infections in this paper.

Beyond absolute cytokine concentrations, disease severity in elderly patients is influenced by additional aspects, including the balance between pro-inflammatory and regulatory signaling, the temporal resolution of the inflammatory response, and underlying comorbidities. Therefore, in severe cases, cytokine levels likely reflect not only viral injury but also coexisting factors such as endothelial dysfunction, secondary infections, and age-related immune remodeling [5,16].

By Day 5, cytokine levels declined substantially in both infections, and differences between groups disappeared. This rapid decline in COVID-19 patients is consistent with Omicron-era findings, in which severe systemic hyperinflammation, and implicitly severe clinical forms, have become less common [30,31]. Regarding FluA, published analyses suggest that while cytokine storms remain a recognized feature of highly pathogenic or pandemic FluA, many seasonal FluA cases follow a more benign course: cytokine levels rise during the acute phase but rapidly decline as viral clearance proceeds [32,33]. The finding also aligns with established concepts of immunosenescence, where innate responses tend to be less sustained in older adults [12,13]. We observed a very low mortality in our study, in accordance with an attenuated inflammatory profile, but this also reduced the discriminative capacity of several cytokines.

A methodological strength of this study is the use of paired cytokine sampling at Day 1 and Day 5, which remains infrequent in comparative viral immunology. Most published cytokine studies rely on single time points, limiting their capacity to capture trajectory or resolution. By characterizing evolving cytokine patterns, our analysis demonstrates that the differences between the groups are primarily evident in the early phase of illness but express rapid convergence thereafter.

The consistently low values of IL-1β and IL-34 in this elderly cohort likely reflect limited systemic expression of these cytokines, rather than technical limitations of the assay. Although IL-34 can be detected in some viral infections, including COVID-19 [34], its circulating levels are typically very low, and, as previously mentioned, immunosenescence further reduces the expression of low-abundance cytokines in older adults [12,13]. In addition, Luminex-based multiplex assays are known to perform less reliably at the lower end of the detection range, especially for analytes that circulate at picogram levels [35]. Importantly, internal assay controls, bead counts, and replicate variability were within limits for the remaining cytokines, indicating that the technical limitations affected only these low-concentration analytes and did not affect the validity of the broader cytokine dataset.

From a clinical standpoint, these findings support the value of early cytokine profiling in elderly patients with acute viral pneumonia. IL-6 remains the most robust early marker of respiratory deterioration, while MCP-1 may identify patients with more favorable immune trajectories. The pathogen-specific differences observed underscore the importance of integrating immunological and clinical information when evaluating risk in older adults. Larger multicenter studies with standardized paired sampling will be essential to confirm the prognostic potential of cytokine dynamics, particularly for MCP-1.

### Study Limitations

This study has several limitations that need to be acknowledged. First, the sample size is relatively small, particularly when stratified by infection type and clinical outcome, which limits the statistical power to detect subtle differences or to perform extensive multivariable adjustment. Second, the inability to reliably detect IL-1β and IL-34 reflects both biological and technical constraints, especially in an elderly population with low-abundance cytokines and the known sensitivity limits of multiplex bead assays; therefore, the absence of signal does not necessarily guarantee the absence of biological relevance. Third, our cohort consists exclusively of older adults (≥60 years) with respiratory failure at admission, which improves internal validity but limits the possibility of extending the findings to younger patients or those with milder disease. Fourth, a majority of patients were unvaccinated for either infection; therefore, vaccine data were unreliable for analysis. Future studies in larger populations with more frequent serial sampling are needed to confirm and extend our findings.

## 4. Materials and Methods

### 4.1. Study Design and Patient Population

We conducted a prospective cohort study at the Clinical Hospital of Infectious Diseases in Cluj-Napoca, Romania. We enrolled patients hospitalized between 10 March 2023 and 31 March 2024 with either confirmed COVID-19 or influenza A. Eligible patients were ≥60 years old, with respiratory symptoms, a peripheral oxygen saturation (SpO_2_) <93% without supplemental oxygen at admission, and consented to participation. Diagnosis of COVID-19 or FluA was established using rapid antigen tests and/or molecular assays, according to WHO case definitions. All patients were tested from a nasopharyngeal swab for both SARS-CoV-2 and FluA. Tests used for diagnosis were: Vitrotrack influenza A + B rapid antigenic test (Vitrotest, Istanbul, Türkiye, 87.2% sensitivity and 94.5% specificity for influenza A), Standard Q COVID-19 antigenic test (SD Biosensor, Suwon, Republic of Korea, 97.12% sensitivity, 100% specificity), GeneXpert-Cepheid@ real-time PCR testing (Cepheid, Sunnyvale, CA, USA, 98.9% sensitivity for influenza A; 98.4% sensitivity for influenza B; 97.5% specificity for influenza A; 99.3% specificity for influenza B; 97% sensitivity and specificity for SARS-CoV-2), DiagCORE-Qiagen@ RT-PCR influenza A and B (QIAGEN, Hilden, Germany, 97.84% sensitivity and 99.45% specificity), NeuMoDxTM RT-PCR SARS-CoV-2 assay (QIAGEN, Hilden, Germany, 100% sensitivity, 98% specificity). The listed diagnostic assays reflect the range of tests used in clinical practice, which were performed according to clinical indication and standard-of-care protocols.

We excluded patients with co-infection with SARS-CoV-2 and influenza A/B, influenza B, with negative testing, or without respiratory symptoms. Based on country-level epidemiological data, all COVID-19 infections during the study period were attributable to the Omicron variant of SARS-CoV-2; no significant shifts in clinical presentation or management occurred, and similarly, FluA cases were caused by antigenically similar strains [36,37]. Case definitions followed WHO recommendations [38,39].

Demographic, clinical, laboratory, and outcome data were extracted from electronic medical records.

Written informed consent was obtained from all participants prior to study inclusion. This study was conducted in accordance with the Declaration of Helsinki and received ethical approval from the Institutional Ethics Committee of the Clinical Hospital of Infectious Diseases, Cluj-Napoca (Approval No. 3420/10 March 2023), and from the Ethics Committee of “Iuliu Hațieganu” University of Medicine and Pharmacy, Cluj-Napoca (Approval No. AVZ 74).

### 4.2. Clinical Data and Outcomes

Parameters were recorded at admission and during hospitalization. For each subject, we extracted variables such as age, sex, length of hospitalization, intensive-care unit (ICU) admission, comorbidities (stratified with age-adjusted Charlson comorbidity index, ACCI), baseline laboratory parameters, radiology, therapeutic decisions, and in-hospital mortality. All patients were treated according to national and international protocols [40,41]. The primary outcome was non-invasive ventilation (NIV) requirement. Secondary outcomes included intensive-care unit admittance and in-hospital mortality. Blood samples were collected on Day 1 (≤24 h after admission) and Day 5, processed within 2 h of collection, and stored at −80 °C until analysis.

### 4.3. Cytokine Quantification

Serum concentrations of IL-1β, IL-6, IL-10, IL-17A, IL-34, MCP-1 (CCL2), and CXCL10 (IP-10) were quantified using Luminex^®^ multiplex immunoassays (R&D Systems/Bio-Techne, Minneapolis, MN, USA) on a FLEXMAP 3D^®^ system (Luminex Corporation, Austin, TX, USA) operated with xPONENT^®^ software version 4.2.1513, using 5-parameter logistic (5-PL) regression models, at the Advanced Center for Medical and Pharmaceutical Research of the G.E Palade University of Medicine, Pharmacy, Science, and Technology of Târgu Mureș, Romania. All assays were performed using LXSAHM-07 cytokine panels. For each analyte, calibration curves were generated using manufacturer-provided standards, with the lowest standard concentration defining the lower end of the calibrated quantification range supported by the assay.

The lowest standard concentrations were in the low pg/mL range and varied by analyte according to the manufacturer’s specifications (19.05 pg/mL for IL-1β; 4.9 pg/mL for IL-6; 5.02 pg/mL for IL-10; 15.39 pg/mL for IL-17A; 93.33 pg/mL for IL-34; 32.43 pg/mL for MCP-1; 1.81 pg/mL for CXCL-10). Concentration values falling below this calibrated range were occasionally reported by the software either as “<lowest standard” or as extrapolated values derived from the fitted standard curve. Extrapolated values below the lowest standard were retained as continuous variables in the dataset, consistent with the 5-PL–based multiplex assays.

In addition to calculated concentrations (pg/mL), net median fluorescence intensity (net MFI) values—representing background-subtracted bead-level signal intensity—were recorded for all analytes and used to support interpretation of low or extrapolated concentrations at the lower end of the assay range. Net MFI values were not analyzed as independent endpoints.

All assay runs included in the final dataset met manufacturer-recommended quality control criteria for bead count, standard curve performance, and goodness-of-fit parameters, as assessed within the xPONENT^®^ software environment.

### 4.4. Statistical Analysis

Data collection and data management were performed with Microsoft Excel, and statistical analyses were performed using the R environment for statistical computing and graphics (version 4.3.2; R Foundation for Statistical Computing, Vienna, Austria). Continuous variables were assessed for normality and are presented as mean (standard deviation) or median with interquartile range (IQR). Categorical variables are reported as frequencies and percentages.

Group comparisons between the COVID-19 and influenza A cohorts were conducted using appropriate parametric (chi-squared test for categorical data) or non-parametric tests (Mann–Whitney U for continuous data, Fisher’s exact test for categorical data) based on data distribution. The Spearman correlation coefficient was used to assess the relationships between pairs of cytokine variables. We used receiver operating characteristic (ROC) analysis to derive data-driven thresholds for each biomarker at Day 1 and Day 5. These cut-offs were not intended as diagnostic classifiers but solely as exploratory criteria to dichotomize cytokine values for multivariate logistic regression. Logistic regression models were applied to explore cytokines as predictors of primary clinical outcomes. Variables exceeding the ROC-derived threshold were categorized as ‘high’, while those below the threshold were categorized as ‘low’. All models were adjusted for infection type (COVID-19 vs. influenza A). For all models, assumptions were checked: the goodness-of-fit with the Hosmer and Lemeshow test the multicollinearity with the variance inflation factor. A two-tailed *p*-value < 0.05 was considered statistically significant.

## Figures and Tables

**Table 1:** Baseline characteristics and comorbidities of patients hospitalized for COVID-19 vs. influenza A.

Variables	COVID-19 (*n* = 39)	Influenza A (*n* = 44)	*p*-Value
Age (years), median (IQR)	79 (73.5–84)	77 (71–81)	0.239
Sex (F), *n* (%)	18 (46.15)	35 (79.55)	0.002
ACCI, median (IQR)	5 (4–7)	5 (4–7.25)	0.562
Length of stay, median (IQR)	8 (6–11)	8 (6–9.25)	0.505
***Comorbidities***			
Active cancer, *n* (%)	3 (7.69)	4 (9.09)	1
Asthma, *n* (%)	2 (5.13)	6 (13.64)	0.272
Atrial fibrillation, *n* (%)	11 (28.21)	9 (20.45)	0.41
Connective tissue disease, *n* (%)	1 (2.56)	1 (2.27)	1
Chronic kidney disease, *n* (%)	5 (12.82)	5 (11.36)	1
Chronic hepatitis, *n* (%)	1 (2.56)	0 (0)	0.47
Congestive heart failure, *n* (%)	14 (35.9)	19 (43.18)	0.499
COPD, *n* (%)	6 (15.38)	15 (34.09)	0.05
Dementia, *n* (%)	6 (15.38)	6 (13.64)	0.821
Diabetes mellitus, *n* (%)	11 (28.21)	12 (27.27)	0.925
Hemiplegia, *n* (%)	5 (12.82)	0 (0)	0.02
Hypertension, *n* (%)	34 (87.18)	39 (88.64)	1
History of myocardial infarction, *n* (%)	5 (12.82)	2 (4.55)	0.245
History of stroke or TIA, *n* (%)	11 (28.21)	7 (15.91)	0.175
Ischemic heart disease, *n* (%)	15 (38.46)	14 (31.82)	0.526
Leukemia, *n* (%)	1 (2.56)	0 (0)	0.47
Obesity, *n* (%)	11 (28.21)	17 (38.64)	0.316
Peptic ulcer disease, *n* (%)	0 (0)	4 (9.09)	0.119
Peripheral vascular disease, *n* (%)	6 (15.38)	5 (11.36)	0.59

IQR—interquartile range; ACCI—age-adjusted Charlson comorbidity index; COPD—chronic obstructive pulmonary disease; TIA—transient ischemic attack; AIDS, lymphoma, and leukemia are criteria in the age-adjusted Charlson comorbidity index, but none of the patients in our groups had these diseases.

**Table 2:** Baseline laboratory parameters and imaging findings in patients hospitalized for COVID-19 vs. influenza A.

Variables	COVID-19 (n = 39)	Influenza A (n = 44)	*p*-Value
**Laboratory findings, median (IQR)**			
Leucocyte ^1^	6.28 (5.42–11.09)	6.91 (5.18–8.81)	0.547
Neutrophils ^1^	5.11 (3.82–8.88)	5.45 (3.88–7.01)	0.578
Lymphocytes ^1^	0.82 (0.59–0.95)	0.86 (0.56–1.16)	0.294
Monocytes ^1^	0.37 (0.24–0.68)	0.4 (0.28–0.56)	0.712
Thrombocytes ^1^	188 (137.5–232)	178 (151–238.5)	0.559
Hemoglobin (g/dL)	12.8 (11.8–13.75)	12.35 (11.9–13.8)	0.975
NLR	7.63 (4.27–12.53)	6.37 (4.37–8.6)	0.214
dNLR	5.43 (2.71–7.33)	3.94 (2.96–5.22)	0.201
PLR	241.12 (176.7–300.48)	204.17 (160.77–287.94)	0.375
SII	1330.59 (744.32–2646.22)	1108.29 (806.96–1543.85)	0.304
SIRI	2.2 (1.68–4.28)	2.51 (1.65–4.15)	1
MLR	0.47 (0.35–0.73)	0.52 (0.37–0.66)	0.931
C-reactive protein(mg/dL)	7.02 (4.07–12.66)	5.78 (2.98–12)	0.608
Radiological appearance *, n (%)			
Ground glass opacities	21 (53.85)	11 (25)	0.007
Consolidation, unilateral	11 (28.21)	13 (29.55)	0.893
Consolidation, bilateral	12 (30.77)	7 (15.91)	0.108
Interstitial pattern	23 (58.97)	32 (72.73)	0.186

1—*10^3^/µL; IQR—interquartile range; NLR—neutrophils-to-lymphocytes ratio; dNLR—derived neutrophils-to-lymphocytes ratio, neutrophils/[leucocytes-neutrophils]; PLR—platelets-to-lymphocytes ratio; SII—systemic inflammation index, neutrophils*platelets/lymphocytes; SIRI—systemic inflammation response index, neutrophils*monocytes/lymphocytes; MLR—monocytes-to-lymphocytes ratio; * Patients exhibited one or more of the radiologic lesions described.

**Table 3:** Outcomes, acute renal failure, and vascular complications of patients hospitalized for COVID-19 vs. influenza A.

Variables	COVID-19 (n = 39)	Influenza A (n = 44)	*p*-Value
Antibiotic treatment, *n* (%)	39 (100)	43 (97.73)	1
Acute respiratory failure, *n* (%)	39 (100)	44 (100)	1
Non-invasive ventilation, *n* (%)	25 (64.1)	34 (77.27)	0.187
Invasive ventilation, *n* (%)	2 (5.13)	2 (4.55)	1
Acute renal failure, *n* (%)	6 (15.38)	14 (31.82)	0.081
Newly diagnosed atrial fibrillation, *n* (%)	11 (28.21)	9 (20.45)	0.41
Pulmonary embolism, *n* (%)	1 (2.56)	1 (2.27)	1
Stroke, *n* (%)	1 (2.56)	0 (0)	0.47
ICU admission, *n* (%)	7 (17.95)	3 (6.82)	0.178
Days of ICU stay, median (IQR)	8 (3–12)	9 (5–23)	0.9
Deceased, *n* (%)	3 (7.69)	2 (4.55)	0.662

IQR—interquartile range; ICU—intensive-care unit.

**Table 4:** Cytokine values on Day 1 and Day 5 in hospitalized patients with COVID-19 or influenza A *.

		Day 1				Day 5				
		Min	Max	Median	*p*	Min	Max	Median	Median Fold Decrease	*p*
**IL-6**	**COVID-19**	2.25	4470.82	12.14	**0.009**	0	160.12	3.22	**3.77**	**0.11**
	**FluA**	0.69	214.36	5.9		0	17.62	1.56	**3.78**	
**IL-10**	**COVID-19**	9.44	404.99	100	**0.44**	6.89	195.65	19.31	**5.17**	**0.27**
	**FluA**	9.64	280.15	65.22		6.89	195.56	14.28	**4.56**	
**IL-17A**	**COVID-19**	2.34	11.83	5.2	**0.5**	0.008	1.86	0.05	**104**	**0.23**
	**FluA**	3.33	12.78	5.2		0.008	10.32	0.02	**260**	
**MCP-1**	**COVID-19**	49.63	2741.46	401.03	**0.1**	72.57	827.67	265.18	**1.51**	**0.69**
	**FluA**	122.26	4654.72	305.65		102.65	2212.52	284.13	**1.07**	
**CXCL10**	**COVID-19**	31.57	1698.6	458.95	**0.39**	33.9	1001.06	78.9	**5.81**	0.75
	**FluA**	55.8	1292.54	364.7		34.34	839.89	85.38	**4.27**	

* All values are represented as pg/mL. *p*-values represent between-group comparisons (COVID-19 vs. FluA) at Day 1 (white background in table)and at Day 5 (blue background in table), respectively (Mann–Whitney U test).

**Table 5:** Receiver operating characteristic classifying the risk for non-invasive ventilation according to measured cytokines at Day 1 and Day 5.

Variable	AUC (95% CI)	Se	Sp	Cut-Off
IL-6 Day 1	0.603 (0.473–0.733)	75	54.24	8.715
IL-6 Day 5	0.552 (0.421–0.68)	50	67.8	4.187
IL-10 Day 1	0.505 (0.357–0.646)	25	91.53	190.82
IL-10 Day 5	0.528 (0.39–0.67)	50.85	62.5	17.097
IL-6/IL-10 Day 1	0.618 (0.484–0.74)	66.67	55.93	0.13
IL-6/IL-10 Day 5	0.542 (0.403–0.684)	58.33	54.24	0.16
Δ IL-6/IL-10	0.523 (0.375–0.668)	72.88	50	−0.052
MCP-1 Day 1	0.564 (0.434–0.707)	67.8	54.17	301.919
MCP-1 Day 5	0.686 (0.552–0.806)	66.1	70.83	251.245
CXCL10 Day 1	0.524 (0.373–0.67)	37.5	76.27	719.467
CXCL10 Day 5	0.575 (0.43–0.706)	64.41	54.17	70.552
IL-17A Day 1	0.526 (0.389–0.664)	79.66	29.17	4.269

AUC—area under curve; Se—sensitivity; Sp—specificity; Δ values represent percentage change from Day 1 to Day 5.

**Table 6:** Multivariate logistic regression models predicting noninvasive ventilation in correlation with disease type and different cytokines.

Variable	OR Adjusted	(95% CI)	*p*
Influenza A vs. COVID-19	0.67	(0.24–1.85)	0.444
IL-6 Day 1 ≥ 8.715	3.02	(1.06–9.53)	0.046
Influenza A vs. COVID-19	0.54	(0.2–1.43)	0.221
IL-6 Day 5 ≥ 4.187	1.28	(0.48–3.42)	0.617
Influenza A vs. COVID-19	0.52	(0.19–1.36)	0.188
Δ IL-6 (increase vs. decrease)	1.8	(0.5–8.6)	0.402
Influenza A vs. COVID-19	0.52	(0.19–1.38)	0.196
IL-10 Day 1 ≥ 190.82	2.95	(0.82–10.8)	0.094
Influenza A vs. COVID-19	0.48	(0.18–1.28)	0.147
IL-10 Day 5 ≥ 17.097	0.61	(0.22–1.62)	0.327
Influenza A vs. COVID-19	0.53	(0.19–1.41)	0.208
Δ IL-6/IL-10 ≥ −0.052 (increase vs. decrease)	0.38	(0.14–1.01)	0.054
Influenza A vs. COVID-19	0.39	(0.13–1.09)	0.081
MCP-1 Day 1 ≥ 301.919	0.32	(0.11–0.88)	0.031
Influenza A vs. COVID-19	0.49	(0.17–1.35)	0.173
MCP-1 Day 5 ≥ 251.245	0.25	(0.09–0.67)	0.008
Influenza A vs. COVID-19	0.5	(0.18–1.31)	0.165
ΔMCP-1 (increase vs. decrease)	1.61	(0.59–4.4)	0.347
Influenza A vs. COVID-19	0.55	(0.2–1.44)	0.222
CXCL10 D1 ≥ 59.969	0.21	(0.01–2.33)	0.213
Influenza A vs. COVID-19	0.53	(0.2–1.38)	0.195
CXCL10 D5 ≥ 70.552	0.55	(0.21–1.47)	0.234
Influenza A vs. COVID-19	0.52	(0.2–1.36)	0.189
ΔCXCL10 (increase vs. decrease)	0.79	(0.04–6.72)	0.845
Influenza A vs. COVID-19	0.53	(0.2–1.37)	0.192
IL-17A D1 ≥ 4.269	0.66	(0.14–3.48)	0.59

High vs. low cytokine categories were defined using ROC-derived thresholds. For Δ variables, “increase” indicates Day 5 ≥ Day 1, and “decrease” indicates Day 5 < Day 1. All models were adjusted for infection type (COVID-19 vs. influenza A).

## Data Availability

The raw data supporting the conclusions of this article can be made available by the authors on request.

## Supplementary Materials

The following supporting information can be downloaded at: https://www.mdpi.com/article/10.3390/ijms27031463/s1.
