# Supplementary material for: Cytokine Dynamics in Severe COVID-19 vs. Influenza A Elderly Patients: A Prospective Comparative Study
Source: Int J Mol Sci. 2026 Feb 1;27(3):1463. doi: 10.3390/ijms27031463 (PMC12898413; doi:10.3390/ijms27031463)
Supplement: Supplementary file 1 [file ijms-27-01463-s001.zip › ijms-4115658-supplementary.pdf]

Supplementary Table S1. Correlation matrices showing associations between percentage changes (Δ%) of cytokines from Day 1 to Day 5 in patients with severe COVID-19

	**Δ IL-6**	**Δ IL-10**	**Δ IL-17A**	**ΔMCP-1**	**ΔCXCL10**
ΔIL-6	-	0.29 (0.069)	0.5 (0.001)	0.52 (< 0.001)	0.39 (0.015)
ΔIL-10	0.29 (0.069)	-	0.54 (< 0.001)	0.12 (0.455)	0.95 (< 0.001)
ΔIL-17A	0.5 (0.001)	0.54 (< 0.001)	-	0.47 (0.003)	0.62 (< 0.001)
ΔMCP-1	0.52 (< 0.001)	0.12 (0.455)	0.47 (0.003)	-	0.22 (0.173)
ΔCXCL10	0.39 (0.015)	0.95 (< 0.001)	0.62 (< 0.001)	0.22 (0.173)	-

Δ values represent percentage change from Day 1 to Day 5: Δ(%) = 100 × (Day 5 – Day 1) / Day 1. *p* values are represented in brackets. Correlations were exploratory and not adjusted for multiple comparisons.

Supplementary Table S2. Correlation matrices showing associations between percentage changes (Δ%) of cytokines from Day 1 to Day 5 in patients with severe influenza A

	**Δ IL-6**	**Δ IL-10**	**Δ IL-17A**	**ΔMCP-1**	**ΔCXCL10**
ΔIL-6	-	0.3 (0.05)	0.39 (0.009)	0.28 (0.061)	0.31 (0.041)
ΔIL-10	0.3 (0.05)	-	0.46 (0.002)	0.35 (0.022)	0.99 (< 0.001)
ΔIL-17A	0.39 (0.009)	0.46 (0.002)	-	0.18 (0.249)	0.44 (0.003)
ΔMCP-1	0.28 (0.061)	0.35 (0.022)	0.18 (0.249)	-	0.37 (0.013)
ΔCXCL10	0.31 (0.041)	0.99 (< 0.001)	0.44 (0.003)	0.37 (0.013)	-

Δ values represent percentage change from Day 1 to Day 5: Δ(%) = 100 × (Day 5 – Day 1) / Day 1. *p* values are represented in brackets. Correlations were exploratory and not adjusted for multiple comparisons.
